# Differential inflammation-mediated function of prokineticin 2 in the synovial fibroblasts of patients with rheumatoid arthritis compared with osteoarthritis

**DOI:** 10.1038/s41598-021-97809-z

**Published:** 2021-09-15

**Authors:** Kentaro Noda, Bianca Dufner, Haruyasu Ito, Ken Yoshida, Gianfranco Balboni, Rainer H. Straub

**Affiliations:** 1 Laboratory of Experimental Rheumatology and Neuroendocrine Immunology, Department of Internal Medicine I, University Hospital Regensburg, Biopark I, Am Biopark 9, 93053 Regensburg, Germany; 2 Division of Rheumatology, Department of Internal Medicine, The Jikei University School of Medicine, Tokyo, Japan; 3 Department of Life and Environmental Sciences, University of Cagliari, Cagliari, Italy

**Keywords:** Rheumatoid arthritis, Osteoarthritis, Neurotrophic factors, Neuroimmunology, Chronic inflammation

## Abstract

Prokineticin 2 (PK2) is a secreted protein involved in several pathological and physiological processes, including the regulation of inflammation, sickness behaviors, and circadian rhythms. Recently, it was reported that PK2 is associated with the pathogenesis of collagen-induced arthritis in mice. However, the role of PK2 in the pathogenesis of rheumatoid arthritis (RA) or osteoarthritis (OA) remains unknown. In this study, we collected synovial tissue, plasma, synovial fluid, and synovial fibroblasts (SF) from RA and OA patients to analyze the function of PK2 using immunohistochemistry, enzyme-linked immunosorbent assays, and tissue superfusion studies. PK2 and its receptors prokineticin receptor (PKR) 1 and 2 were expressed in RA and OA synovial tissues. PKR1 expression was downregulated in RA synovial tissue compared with OA synovial tissue. The PK2 concentration was higher in RA synovial fluid than in OA synovial fluid but similar between RA and OA plasma. PK2 suppressed the production of IL-6 from TNFα-prestimulated OA-SF, and this effect was attenuated in TNFα-prestimulated RA-SF. This phenomenon was accompanied by the upregulation of PKR1 in OA-SF. This study provides a new model to explain some aspects underlying the chronicity of inflammation in RA.

## Introduction

Rheumatoid arthritis (RA) is a chronic inflammatory disease in synovial joints characterized by synovial cell proliferation and extensive inflammatory cell infiltration (pannus). RA patients show immunological abnormalities, including anti-citrullinated protein/peptide antibodies and rheumatoid factor^1^. The main symptoms of RA include tenderness and swelling in multiple joints due to inflammation, and in patients with advanced disease, long-term inflammation leads to cartilage and bone destruction, resulting in joint deformities. The secondary symptoms are associated with chronic inflammation (depression, appetite loss, and insomnia) and impaired health-related quality of life^2^.

Factors that regulate the neuroendocrine pathways connecting the central nervous system and peripheral tissues, such as hormones, neuropeptides, and sympathetic and sensory nerves, are associated with RA symptoms^3^, including morning stiffness^4^ and pain^5^. Additionally, these factors often play a role in local inflammation in arthritis as their receptors are expressed in synovial tissue. For instance, increased peripheral metabolism of sex hormones is observed in inflamed synovial tissue^6–8^, and a thyroid hormone network exists in synovial tissue in patients with osteoarthritis (OA) and RA^9^. Furthermore, substance P released from sensory nerve fibers plays a proinflammatory role by recruiting leukocytes, promoting their plasma extravasation^10^, and stimulating the secretion of proinflammatory cytokines from synovial fibroblasts (SF) in synovial tissue^11^. In this study, we aimed to elucidate new neuroendocrine pathways, focusing on prokineticin 2 (PK2).

PK2 is a secreted protein with a five-disulfide-bridged motif termed a colipase fold and is involved in regulating neuroendocrine pathways^12^. Two types of G-protein-coupled receptors, prokineticin receptor 1 (PKR1) and prokineticin receptor 2 (PKR2)^13^, have been identified to interact with PK2, and both receptors couple to Gαq, Gαs, and Gαi proteins, indicating that PKRs activate multiple signaling pathways^14^. PK2 is expressed in the brain, testis, adrenal gland, uterus, intestine, liver, bone marrow, and blood cells^15,16^. PKR1 is predominantly distributed in peripheral tissue, and PKR2 is mainly distributed in brain tissue^15,17^.

**Figure 1:**
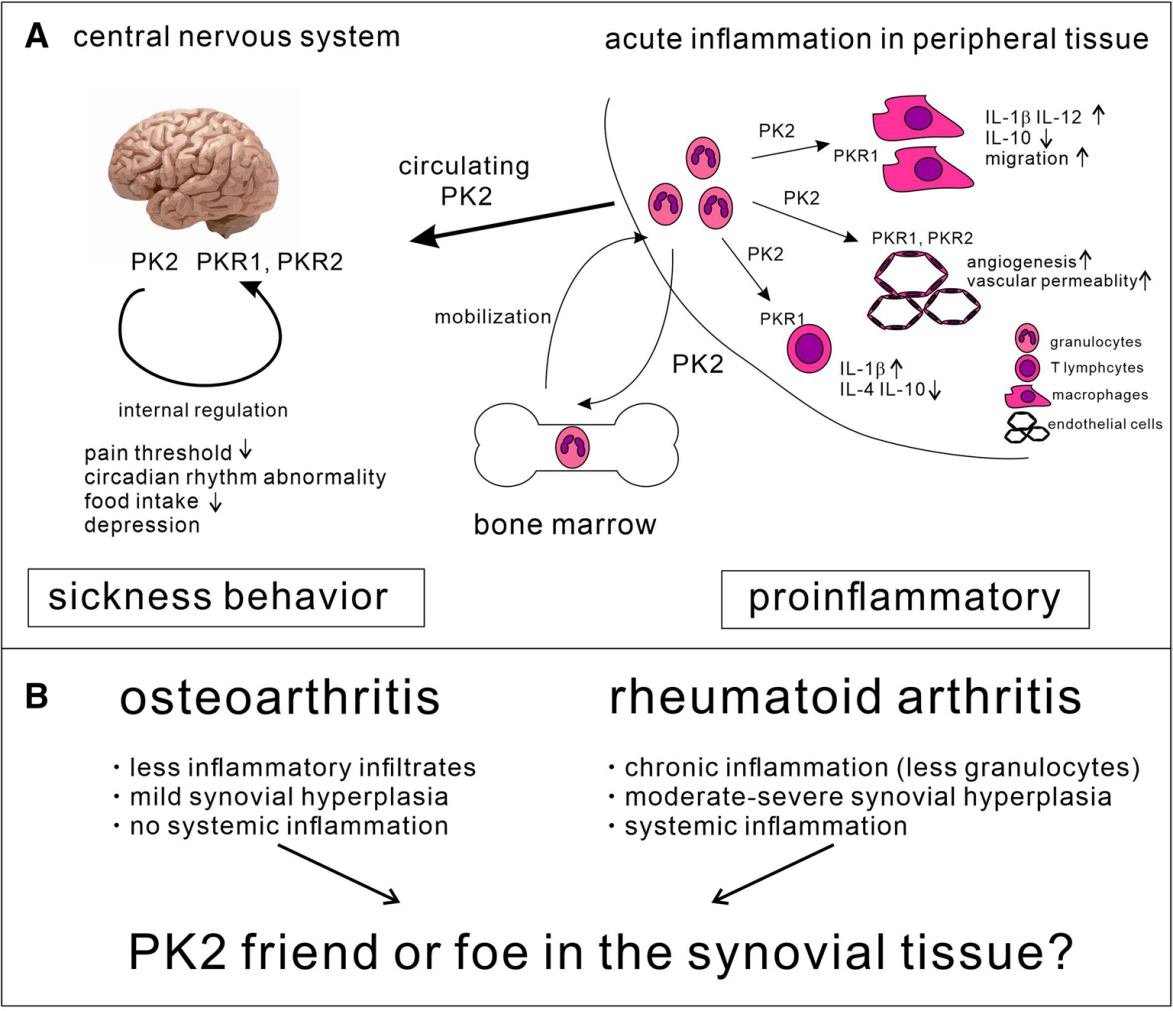
PK2 as a regulator of inflammation and sickness behaviors. (**A**) In acute inflammatory tissue, PK2 is secreted by infiltrating granulocytes. Secreted PK2 mobilizes granulocytes from the bone marrow, promotes the production of proinflammatory cytokines from macrophages and T cells through PKR1, and increases angiogenesis and vascular permeability through PKR1 and PKR2, indicating that PK2 acts as a proinflammatory factor. Secreted PK2 reaches the central nervous system through the bloodstream. It regulates the pain threshold, circadian rhythm, food intake, and mood status through PKR1 and PKR2, indicating that PK2 promotes sickness behaviors under inflammatory conditions. However, in the synovial tissue of rheumatoid arthritis, the most influential cells are synovial cells, not granulocytes (**B**). Therefore, the effect of PK2 in rheumatoid arthritis and osteoarthritis may be different from that in acute inflammatory tissue. *PK2* prokineticin 2, *PKR1*, prokineticin receptor 1, *PKR2* prokineticin receptor 2, *IL* interleukin.

PK2 has multiple physiological functions, including the regulation of neurogenesis^18^, angiogenesis^19^, pain threshold^20,21^, mood^22,23^, circadian rhythm^24^, ingestive behaviors^25–27^, and energy expenditure^28^ (Fig. 1A). PK2 also acts as a proinflammatory factor for inflammatory cells^29,30^. For example, PK2 induces the migration and proinflammatory phenotype of mouse macrophages through the production of interleukin (IL)-1 and inhibition of IL-10^31^, mobilizes neutrophils to the site of inflammation^32^, and suppresses the production of IL-10 and IL-4 in mouse splenocytes through the PKR1 pathway^33^.

The PK2 concentration in the peripheral blood of patients with inflammatory diseases, such as multiple sclerosis^34^ and psoriasis^35^, is increased compared with healthy controls. Taking these factors into consideration, the mechanisms underlying the symptoms and pathogenesis of arthritis appear to be similar to the processes regulated by PK2. However, the association between PK2 and the pathogenesis of RA has not yet been elucidated in patients. We previously showed that PK2 expression was upregulated in the joints of mice with collagen-induced arthritis (CIA)^36^, and the administration of a PKR antagonist attenuated mouse CIA^37^. Several granulocytes are present in the synovial tissue of mice with CIA^38^, whereas most of the influential cells in RA synovial tissue during the chronic phase are synovial cells^39^. Therefore, the function of PK2 in RA synovial tissue may be different compared with acute inflammatory tissue in mice (Fig. 1B).

Based on the described aspects of PK2, we hypothesized that PK2 is associated with the pathogenesis of RA and that the role of PK2 in RA is different from that in OA.

## Materials and methods

### Patients

We conducted a study of 67 patients (19 men, 48 women; mean age = 62.6 ± 10.6 years) with established RA (according to the American College of Rheumatology/European League Against Rheumatism criteria^40^) and 79 patients (31 men, 48 women; mean age 68.0 ± 8.07 years) with OA. All patients underwent total knee arthroplasty in the Department of Orthopedic Surgery, and peripheral blood, synovial fluid, and synovial tissue samples were obtained. The C-reactive protein concentration in RA and OA patients was 15.8 ± 26.7 mg/l and 2.18 ± 3.02 mg/l, respectively, indicating significantly reduced systemic inflammation in OA patients compared with RA patients. Among RA patients, the treatments received included glucocorticoids (51), methotrexate (34), biologics (13), leflunomide (7), and sulfasalazine (3). Among OA patients, one patient received glucocorticoids. We performed this study according to the Helsinki Declaration of 1975, as revised in 1983. Approval for this study was obtained from the Ethics Committee of the University of Regensburg (approval number 15-1 01-021). All patients understood the purpose of the study and provided informed consent.

### Reagents

Human recombinant PK2 (#100-46), tumor necrosis factor α (TNFα) (300-01A), IL-1β (#200-01B), and transforming growth factor β (TGFβ) (#100-21C) were obtained from Peprotech (Rocky Hill, NJ, USA). The PKR1 and PKR2 antagonist^41^ [PKRA7 (508942)] was obtained from Merck (Darmstadt, Germany), and the PKR1 antagonist (PC-7)^42^ was generated by one of the authors of the current study (G.B.).

### Synovial tissue and SF preparation

Synovial tissue samples from patients with RA and OA were obtained immediately after opening the knee joint capsule. Synovial tissue pieces up to 9 cm^2^ were excised. Part of the tissue was minced and treated with Liberase TM (#05401127001, Roche Diagnostics, Mannheim, Germany) at 37 °C for 1 h on a shaking platform. The resulting suspension was filtered (70 μm) and centrifuged at 1600 rpm for 10 min. The pellet was then treated with erythrocyte lysis buffer (20.7 g NH_4_Cl, 1.97 g NH_4_HCO_3_, 0.09 g EDTA and 1 L H_2_O) for 8 min and recentrifuged at 1600 rpm for 10 min. The pellet was resuspended in RPMI-1640 (Sigma Aldrich, St. Louis, MO, USA) with 10% fetal calf serum (FCS). After overnight incubation, cells were supplemented with fresh medium. The culture medium used was RPMI-1640 without phenol red (Sigma Aldrich, St. Louis, MO, USA) supplemented with 10% FCS, 4 mM l-glutamine (Sigma Aldrich, St. Louis, MO, USA), 10 mM HEPES (Sigma Aldrich, St. Louis, MO, USA), 100 U/ml penicillin, 100 μg/ml streptomycin (Sigma Aldrich, St. Louis, MO, USA), and 10 μg/ml ciprofloxacin (Fresenius Kabi, Bad Homburg, Germany). Passage 4–8 SF were used for experiments. The remaining part of the collected tissue was used for superfusion and immunohistochemistry experiments.

### Synovial tissue superfusion

One piece of ~ 16 mm^2^ fresh synovial tissue from patients was loaded into a superfusion chamber (80 μl), as described previously^43^. Then, superfusion was performed for 2 h at 37ºC at a flow rate of 66 μl/min with serum-free culture medium. The superfusate was collected at 2 h and used for enzyme-linked immunosorbent assay (ELISA).

### Histological analysis

The expression of PK2, PKR1, and PKR2 in synovial tissues and SF from patients with OA and RA was analyzed by immunohistochemistry. For synovial tissue staining, the tissues were fixed in 3.7% formalin and embedded in paraffin. The paraffin block was sectioned into 6–8-μm slices and used for staining. The sections were incubated in Dako Target Retrieval Solution (S1699, Agilent, Santa Clara, CA, USA) at 90ºC for 1 h to activate the antigens. Endogenous peroxidase was inactivated with 3% H_2_O_2_ in methanol. The sections were then blocked with 10% normal goat serum, 10% bovine serum albumin (BSA), and 10% FCS for 1 h and incubated with primary antibodies (rabbit anti-PK2 polyclonal antibody [bs-5784R, 2.5 μg/ml] from Bioss antibodies Woburn, MA, USA; rabbit anti-PKR1 [NBP1-83337, 2 μg/ml] and anti-PKR2 polyclonal antibody [NBP1-92290, 1 μg/ml] from Novus Biologicals via Bio-Techne, Wiesbaden, Germany) overnight at 4 °C. Subsequently, the sections were incubated with polyclonal goat anti-rabbit immunoglobulins/HRP (P0448, 0.5 μg/ml) from Agilent, Santa Clara, CA, USA, for 1 h at room temperature. The color was then developed by incubation with ImmPACT DAB (SK-4105) from Vector laboratories, CA, USA, for 10 min at room temperature. The sections were finally counterstained with hematoxylin.

SF from OA and RA patients were cytospun and fixed with cold acetone. The cytospin preparations were blocked with 10% normal goat serum and 10% BSA and then incubated with primary antibodies (rabbit anti-PK2 polyclonal antibody [ab76747, 5 μg/ml] from Abcam, Cambridge UK; rabbit anti-PKR1 polyclonal antibody [NBP1-83337, 1 μg/ml] from Novus Biologicals via Bio-Techne, Wiesbaden, Germany; and mouse anti-PKR2 monoclonal antibody [sc-365696, 4 μg/ml] from Santa Cruz Biotechnology, Heidelberg, Germany) overnight at 4ºC. Subsequently, cytospins were incubated with an Alexa Fluor 488-labeled secondary antibody (A-11070, 2 μg/ml) from Thermo Fisher, Schwerte, Germany. The sections were finally counterstained with 4',6-Diamidine-2'-phenylindole dihydrochloride (DAPI). Control experiments were performed with isotype antibodies instead of the primary antibody.

The sections were examined under a microscope (BX-61, Olympus, Tokyo, Japan). A semiquantitative scoring system was used to analyze the expression of PK2, PKR1, and PKR2 in synovial tissues. PK2-, PKR1-, and PKR2-positive cells in the lining and sublining layer were counted in 5 sequential fields per sample under a high-power field (× 400) by two experienced rheumatologists (KN and HI) in a blinded manner. Positive staining was determined based on the comparison between PK2-, PKR1-, and PKR2-stained sections and isotype-stained samples. The consensus of two researchers was used as the final result in the analysis. The percent positivity was calculated and graded on a scale of 0–4: 0 = no stained cells, 1 = 0–25% stained cells, 2 = 26–50% stained cells, 3 = 51–75% stained cells, and 4 = 76–100% stained cells^44^.

### Cell-based ELISA

To study the cellular expression of PK2, PKR1, and PKR2 under proinflammatory conditions, 1 × 10^4^ cells per well were seeded in a 96-well plate and stimulated with TNFα (10 ng/ml), IL-1β (200 pg/ml), and TGFβ (10 ng/ml) for 24 and 48 h. Then, cells were fixed with 3.7% formalin (for PK2) for 20 min or cold methanol for 10 min (for PKR1 and PKR2). Formalin-treated cells were permeabilized and blocked with 0.1% Triton-X and 1% BSA in phosphate-buffered saline (PBS) for 1 h. Methanol-treated cells were blocked with 1% BSA in PBS for 1 h. After blocking, cells were incubated with primary antibodies overnight at 4 °C. The antibodies and concentrations used were the same as those described above for immunohistochemistry in SF. Cells were incubated with a polyclonal HRP-conjugated goat anti-rabbit secondary antibody (#32260, 0.5 μg/ml for PKR1 and PKR2, 0.25 μg/ml for PK2) from Thermo Fisher, Schwerte, Germany, for 1 h at room temperature and visualized with 1-step Ultra TMB Substrate Solution (#34029 from Thermo Fisher, Schwerte, Germany). After stopping the reaction with 2 M sulfuric acid, the optical density was determined using a Biorad imark™ microplate reader (Bio-rad, München, Germany). To study the phosphorylation of nuclear factor kappa B (NFκB) p65 after stimulation with PK2 under proinflammatory conditions, 1 × 10^4^ cells per well were seeded in a 96-well plate, prestimulated with PK2 (10^–11^ M) for 1 h, and then stimulated with TNFα (10 ng/ml) for 0, 5, 15, 30, 60, and 120 min. Cells were fixed with 3.7% formalin for 20 min and then permeabilized and blocked with 0.3% Triton-X and 1% BSA in PBS for 1 h. After blocking, cells were incubated with an anti-phospho-NFκB p65 (Ser536) rabbit monoclonal antibody (#3033, 1:200) from Cell Signaling Technology, Danvers, MA, USA, overnight at 4ºC. Cells were incubated with a polyclonal HRP-conjugated goat anti-rabbit secondary antibody (2.5 μg/ml) from Thermo Fisher for 1 h at room temperature and visualized with 1-step Ultra TMB from Thermo Fisher. After stopping the reaction with 2 M sulfuric acid, the optical density was determined using a Bio-rad imark™ microplate reader (Bio-rad). Thirteen cell lines (RA, 6 and OA, 6–7) were used for PK2, PKR1, and PKR2 cell-based ELISAs, and 14 cell lines (RA, 6 and OA, 5–8) were used for phospho-NFκB p65 cell-based ELISAs. Each cell line was derived from a different patient. Cell-based ELISAs were performed with 2–3 different cell lines simultaneously, and experiments were repeated two or three times with different cell lines.

### Stimulation of SF

To study the effect of PK2 on SF under proinflammatory conditions, 1 × 10^4^ cells per well were seeded in a 96-well plate, prestimulated with TNFα (10 ng/ml) in RPMI-1640 medium containing 2% FCS for 48 h, then stimulated with medium containing the respective compounds (PC-7 at 1 μM, PKRA7 at 2 μM, or 0.1% DMSO as a control) and PK2 at concentrations from 10^–11^ M to 10^–14^ M. After 24 h, cell culture media were collected and used for ELISAs.

### ELISAs

IL-6, matrix metalloproteinase-3 (MMP-3), osteoprotegerin (OPG), and tissue inhibitor of metalloprotease-1 (TIMP-1) levels in cell culture media were measured using a standard quantitative sandwich ELISA following the manufacturer’s protocol (for IL-6, BD OptEIA, BD Biosciences, Heidelberg, Germany; for MMP-3, OPG, and TIMP-1, DuoSet ELISA, R&D Systems, Minneapolis, MN, USA). PK2 levels in the plasma, synovial fluid, and superfusate from patients with OA and RA were measured using an ELISA kit from Cloud-Clone Corp., Katy, TX, USA, following the manufacturer’s protocol.

### MTT assays, migration assays, chemotactic assays, and animal experiments

These methods are described in the Supplementary Methods.

### Statistical analysis

All data are presented as the mean ± SD. Box plots demonstrate the 10th, 25th, median, 75th, and 90th percentiles. The Mann–Whitney U test was used for two-group comparisons, and the Wilcoxon signed-rank test was used for pairwise comparisons. A one-sample Wilcoxon signed-rank test (when data did not follow a normal distribution) or one-sample t-test (when data were normally distributed) was used to compare the expression of PK2, PKR1, and PKR2 (% of control) in cell-based ELISAs or the concentration of IL-6, MMP-3, TIMP-1, and OPG (% of control) in ELISAs with a fixed population control level of 100%. Spearman rank correlation was used to analyze the correlation between plasma and synovial fluid levels in OA and RA patients. For comparisons between the DMSO control and PC-7 or PKRA7 group in ELISAs, a two-way ANOVA followed by the Bonferroni post hoc test was used. These analyses were conducted with SigmaPlot V.13 (Systat Software, Erkrath, Germany) and GraphPad Prism version 4.0 (GraphPad Software, San Diego, CA, USA). Statistical significance was set at p < 0.05.

## Results

### PK2, PKR1, and PKR2 expression in OA and RA synovial tissue

**Figure 2:**
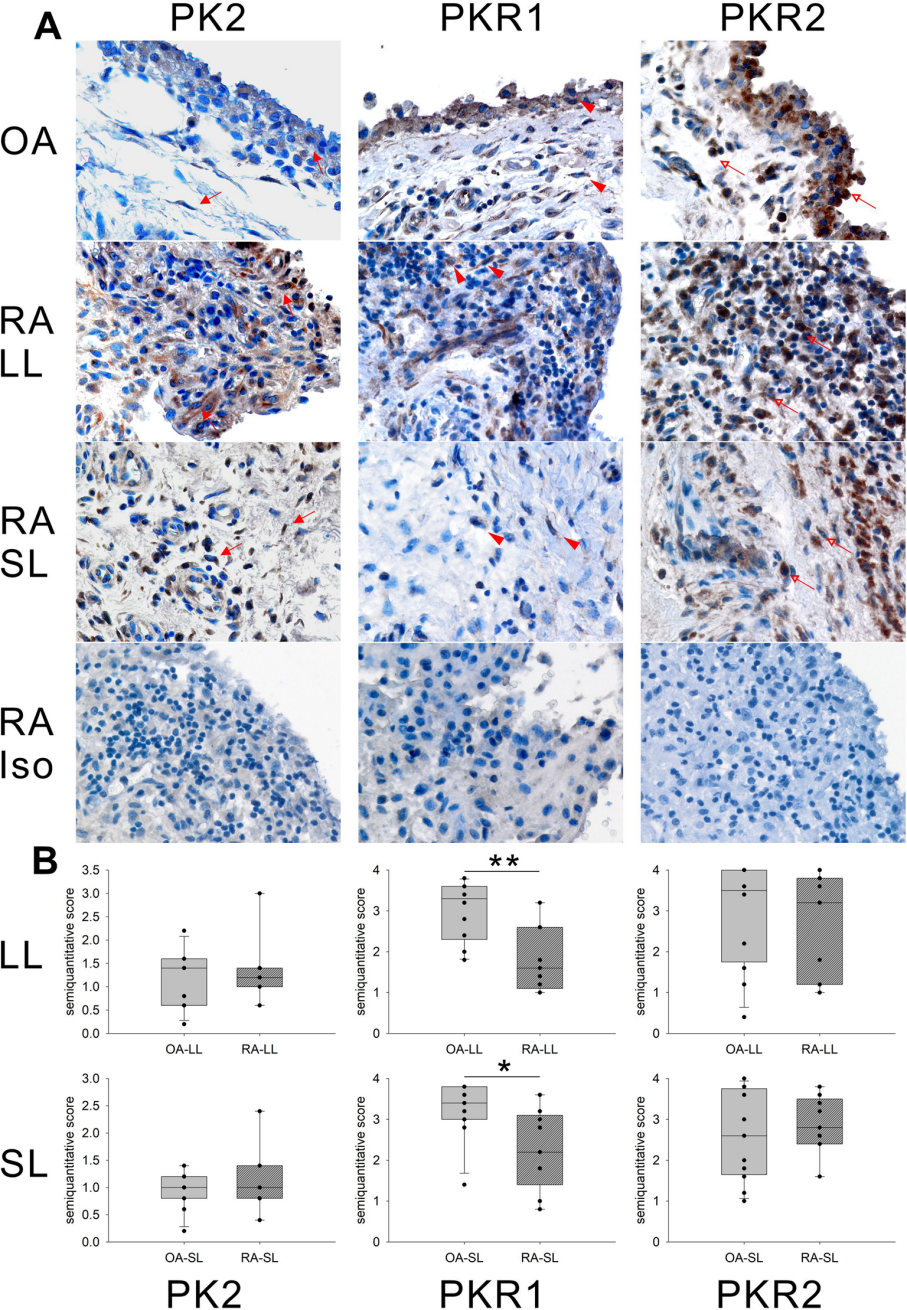
PK2, PKR1, and PKR2 expression in OA and RA synovial tissues. (**A**) Immunostaining of PK2 (left panel), PKR1 (middle panel), and PKR2 (right panel) proteins in synovial tissue from OA and RA patients. Brown staining indicates PK2- (red arrow), PKR1- (red arrowhead), or PKR2 (red open headed arrow)-positive cells (× 400) Staining with an isotype control as the primary antibody in RA synovial tissue is also demonstrated. (**B**) Semiquantitative results of PK2, PKR1, and PKR2 expression. Semiquantification was performed using a scoring method (0–4) in the lining and sublining layers of synovial tissue. Data were presented as box plots, in which the boxes demonstrate the 25th and 75th percentiles, the lines within the boxes demonstrate the median, and the lines outside the boxes demonstrate the 10th and 90th percentiles. N = 8–10. For statistical analysis, the Mann–Whitney test was used. *p < 0.05, **p < 0.01. *PK2* prokineticin 2, *PKR1* prokineticin receptor 1, *PKR2* prokineticin receptor 2, *OA* osteoarthritis, *RA* rheumatoid arthritis, *Iso* isotype control, *SL* sublining layer, *LL* lining layer.

In previous studies, we showed the presence of PK2, PKR1, and PKR2 proteins in the synovial tissue of mice with CIA^36,37^. Therefore, we first examined PK2, PKR1, and PKR2 expression in OA and RA synovial tissue (Fig. 2). Few polymorphonuclear (PMN) cells were observed in OA and RA synovial tissues. A proportion of mononuclear cells showed positive PK2 expression in these tissues (Fig. 2A: arrow), and there was no significant difference in the PK2 positivity rate in the lining and sublining layers between OA and RA tissues (Fig. 2B). Most mononuclear cells in OA tissues and a proportion of mononuclear cells in RA tissues were positive for PKR1 (Fig. 2A: arrowhead), and the PKR1 positivity rates in the OA lining and sublining layers were significantly higher than those in RA. In addition, most mononuclear cells in OA and RA synovial tissues exhibited positive PKR2 expression (Fig. 2A: open-headed arrow), and there was no significant difference in the positivity rate in the lining and sublining layers between OA and RA tissues. In synovial tissues, PK2-, PKR1-, and PKR2-positive mononuclear cells included SF, synovial macrophages, and/or inflammatory cells (except for PMN cells). Collectively, our data demonstrate that PK2, PKR1, and PKR2 were expressed in RA and OA tissues. In addition, PKR1 expression in RA synovial tissue was downregulated compared with that in OA synovial tissue.

### PK2, PKR1, and PKR2 expression in OA- and RA-SF

**Figure 3:**
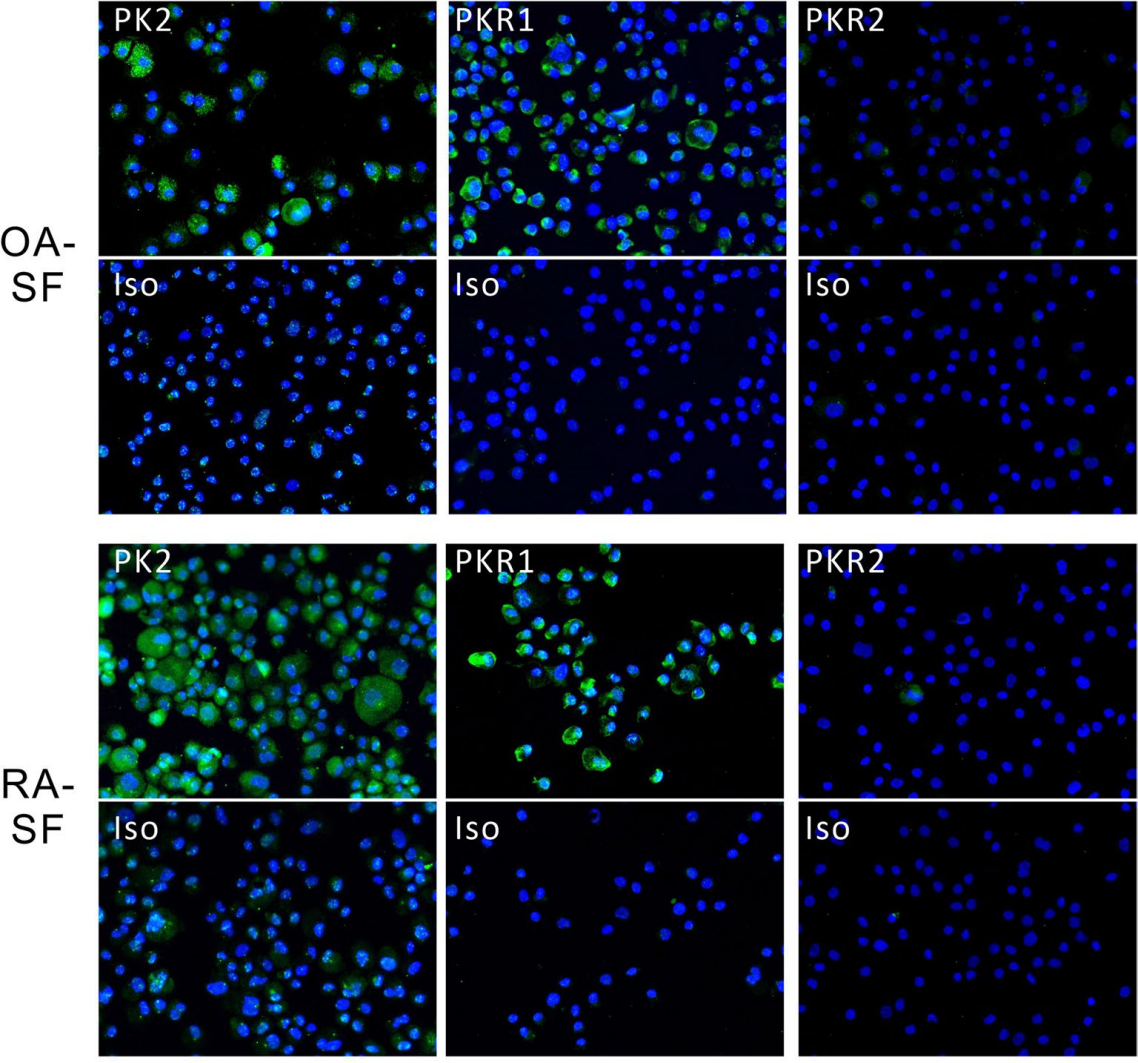
Immunofluorescent staining of PK2, PKR1, and PKR2 proteins in OA- and RA-SF. Cells were subjected to cytospin. Green staining indicates PK2- (left panel), PKR1- (middle panel), or PKR2- (right panel) positive cells (× 200). Stained sections with an isotype control as the primary antibody are also demonstrated. N = 5. *PK2* prokineticin 2, *PKR1* prokineticin receptor 1, *PKR2* prokineticin receptor 2, *OA* osteoarthritis, *RA* rheumatoid arthritis, *SF* synovial fibroblasts.

Next, we examined PK2, PKR1, and PKR2 expression levels in SF collected from OA and RA synovial tissues (Fig. 3). Positive expression of PK2 and PKR1 was observed in OA- and RA-SF (Fig. 3, left and middle panels), whereas minimal PKR2 expression was detected in OA- and RA-SF (Fig. 3, right panel). PK2 staining showed a diffuse fine granular pattern in the cytoplasm. PKR1 staining was mainly localized in the cytoplasmic membrane.

### Modulation of PK2, PKR1, and PKR2 expression in OA- and RA-SF under proinflammatory conditions

**Figure 4:**
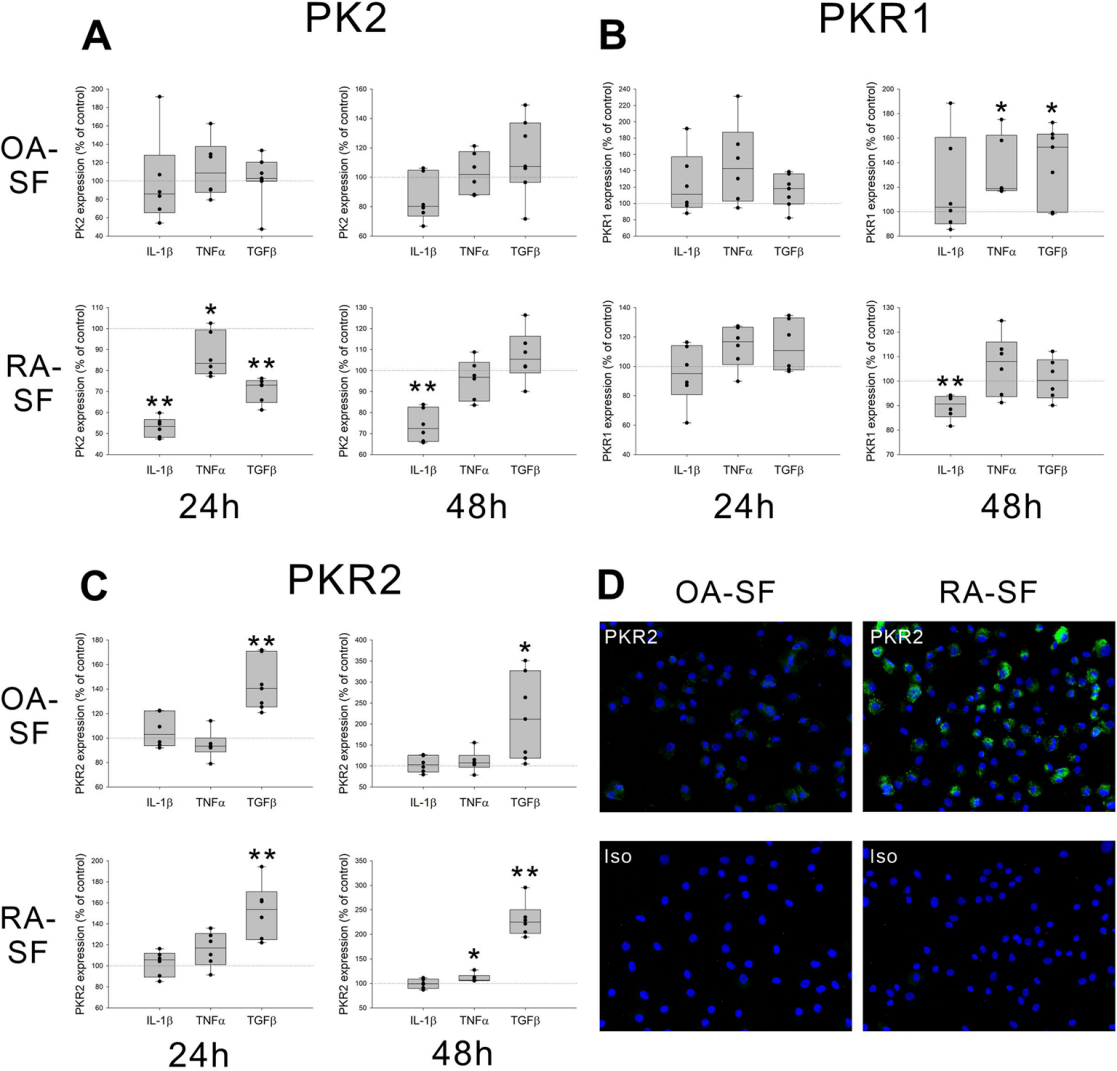
Modulation of PK2, PKR1, and PKR2 expression in OA- and RA-SF under proinflammatory conditions. PK2 (**A**), PKR1 (**B**), and PKR2 (**C**) proteins in OA- and RA-SF were examined by cell-based ELISAs at 24 and 48 h after stimulation with IL-1β (200 pg/ml), TNFα (10 ng/ml), and TGFβ (10 ng/ml). (**D**) Immunofluorescent staining of PKR2 in OA- and RA-SF at 48 h after stimulation with TGFβ. N = 6–7. Green staining indicates PKR2-positive cells (× 200). Staining with an isotype control as the primary antibody is also given. Data are shown as box plots, the description of which is given in the legend to Fig. 2. Each dot in the figure represents a different cell line derived from a different patient. For statistical analysis, the one-sample Wilcoxon signed-rank test (when data did not follow a normal distribution) or the one-sample t-test was used (when data were normally distributed). *p < 0.05, **p < 0.01. *PK2* prokineticin 2, *PKR1* prokineticin receptor 1, *PKR2* prokineticin receptor 2, *OA* osteoarthritis, *RA* rheumatoid arthritis, *SF* synovial fibroblasts, *IL-1β* interleukin-1β, *TNFα* tumor necrosis factor α, *TGFβ* transforming growth factor β, *Iso* isotype control.

Generally, synovial tissue in RA patients is continuously exposed to a variety of proinflammatory cytokines, whereas synovial tissue in OA patients is exposed to these factors under certain conditions. Therefore, we examined the change in PK2, PKR1, and PKR2 expression following stimulation with TNFα, IL-1β, and TGFβ in OA- and RA-SF using cell-based ELISAs (Fig. 4). PK2 expression in OA-SF was not influenced by IL-1β, TNFα, or TGFβ at 24 and 48 h after stimulation. Moreover, PK2 expression in RA-SF was downregulated at 24 h after stimulation with IL-1β, TNFα, and TGFβ, and this downregulation persisted for 48 h after stimulation with IL-1β only (Fig. 4A). IL-1β, TNFα, and TGFβ had no effect on PKR1 expression in OA- and RA-SF at 24 h after stimulation. However, PKR1 expression was upregulated at 48 h after stimulation with TNFα and TGFβ in OA-SF and downregulated at 48 h after stimulation with IL-1β in RA-SF (Fig. 4B). PKR2 expression in OA- and RA-SF was upregulated at 24 and 48 h after stimulation with TGFβ and 48 h after stimulation with TNFα in RA-SF (Fig. 4C). Similar to the results of cell-based ELISAs, positive PKR2 expression was detected by immunohistochemistry in OA- and RA-SF after stimulation with TGFβ (Fig. 4D). These data indicate that the expression of PKR1 under proinflammatory conditions was inversely regulated in OA- and RA-SF, and these findings correspond to the immunohistochemistry results in OA and RA synovial tissues.

### PK2 concentration in plasma, synovial fluid, and superfusate in patients with OA and RA

**Figure 5:**
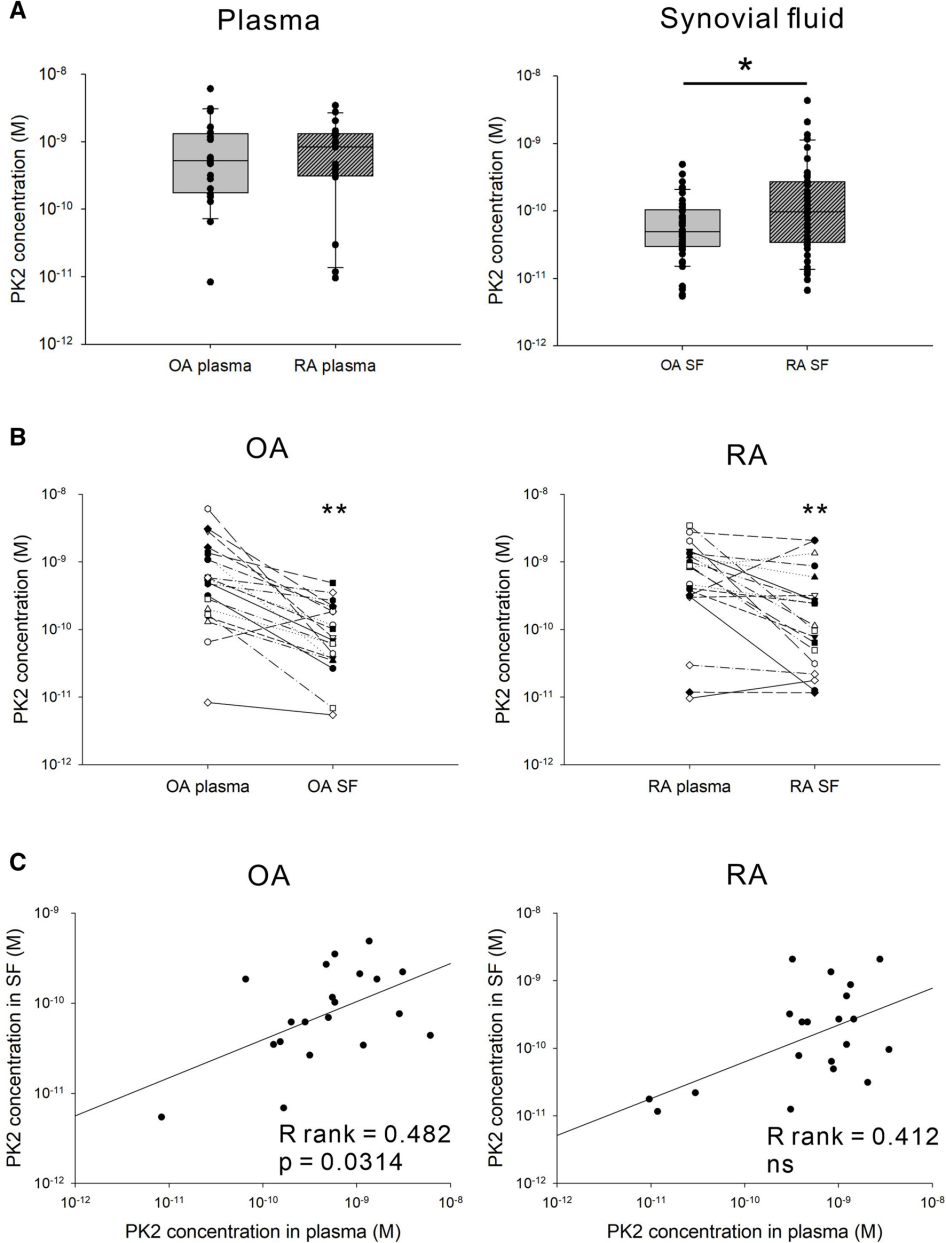
The PK2 concentration in plasma and synovial fluid from OA and RA patients. (**A**) Comparison of plasma (N = 20) and synovial fluid (N = 50) PK2 concentrations between OA and RA patients. Data were presented as box plots, the description of which is given in the legend to Fig. 2. For statistical analysis, the Mann–Whitney test was used. *p < 0.05, **p < 0.01. (**B**) Comparison of PK2 concentrations between plasma and synovial fluid in OA and RA patients. N = 20. For statistical analysis, the Wilcoxon signed-rank test was used. (**C**) Correlation of PK2 concentrations between plasma and synovial fluid in OA and RA patients. N = 20. The linear regression line, Spearman rank correlation coefficient (R rank), and respective p-value are given. *PK2* prokineticin 2, *SF* synovial fluid, *OA* osteoarthritis, *RA* rheumatoid arthritis, *NS* not significant.

The mean plasma PK2 concentration in patients with OA and RA was 1.07 ± 1.47 × 10^–9^ M and 0.97 ± 0.92 × 10^–9^ M, respectively, and was not statistically different between the patient groups (Fig. 5A, left panel). The mean synovial fluid PK2 concentration in patients with OA and RA was 8.36 ± 1.31 × 10^–11^ M and 3.50 ± 7.37 × 10^–10^ M, respectively, and was significantly higher in RA patients than in OA patients (Fig. 5A, right panel).

The superfusate PK2 concentration was below the detection limit of the ELISA Kit. The superfusate concentration is the concentration of interstitial fluid^45^. Therefore, this result indicates that PK2 acts at low concentrations in synovial tissue. In a direct pairwise comparison, the PK2 concentration was substantially lower in synovial fluid than in plasma in patients with OA and RA (Fig. 5B). Moreover, the PK2 concentration in plasma was significantly correlated with that in synovial fluid in OA patients but not in RA patients (Fig. 5C). These results indicate that PK2 in the synovial fluid was mainly from plasma (blood exudate) in OA patients but produced locally in the synovial cavity in addition to blood exudate in RA patients.

### PK2 has an anti-inflammatory effect in OA-SF but not RA-SF

**Figure 6:**
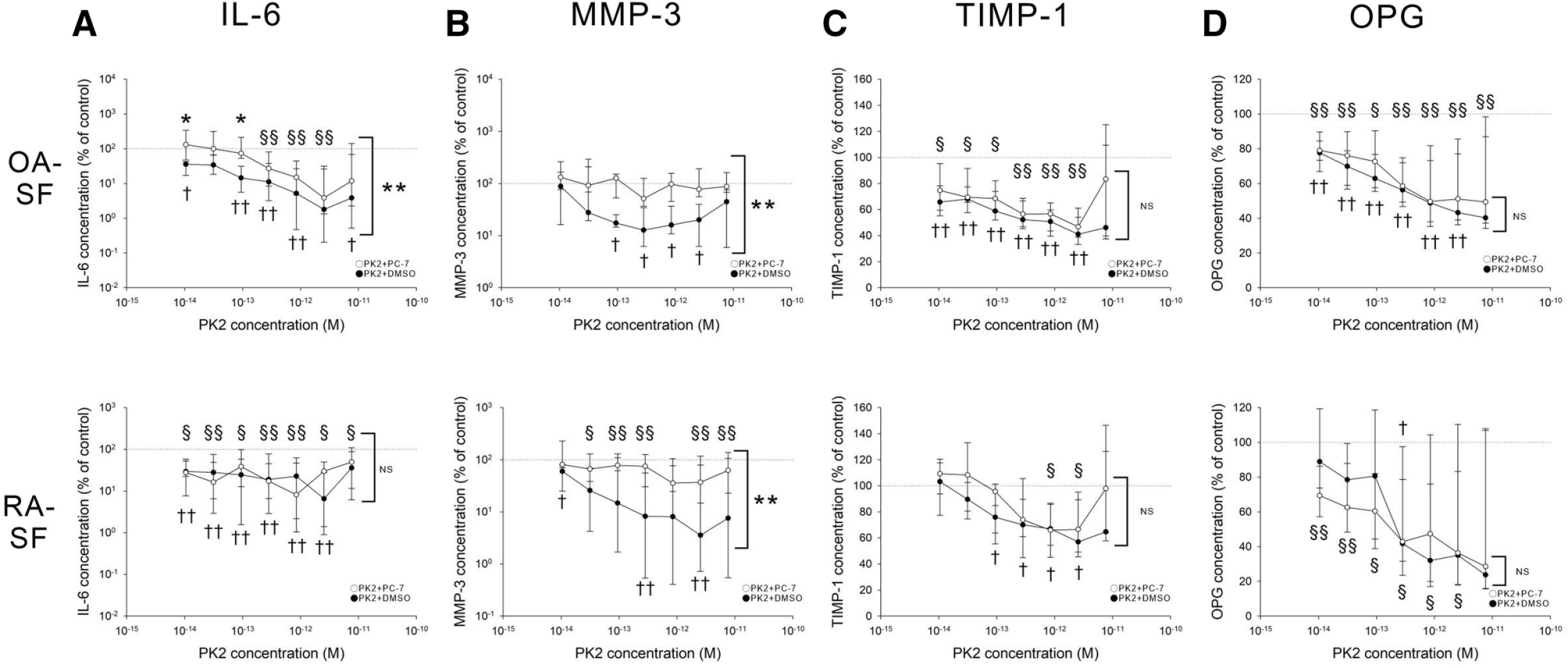
Influence of PK2 and PC-7 (or DMSO) on IL-6 (**A**), MMP-3 (**B**), TIMP-1 (**C**), and OPG (**D**) production from TNFα-prestimulated OA- and RA-SF. The dotted line indicates the control level of 100% (TNFα without PK2). All data are given as the median (25th percentile, 75th percentile). N = 8. In comparisons between DMSO and PC-7 groups, a two-way ANOVA followed by the Bonferroni post hoc test was used (*p < 0.05, **p < 0.01). In comparisons with a control level of 100%, the one-sample Wilcoxon signed-rank test was used (compared with the control in the PC-7 group: ^§^p < 0.05 and ^§§^p < 0.01; compared with the control in the DMSO group: ^†^p < 0.05 and ^††^p < 0.01). *PK2* prokineticin 2, *DMSO* dimethylsulfoxide, *OA* osteoarthritis, *RA* rheumatoid arthritis, *SF* synovial fibroblasts, *IL-6* interleukin-6, *TNFα* tumor necrosis factor α, *MMP-3* matrix metalloproteinase 3, *TIMP-1* tissue inhibitor of metalloproteinase 1, *OPG* osteoprotegerin, *NS* not significant, *ANOVA* analysis of variance.

Based on the results of PK2 ELISAs in plasma, synovial fluid, and superfusate, we predicted that PK2 is present at a low concentration of less than 10^–11^ M in synovial tissue. Because the expression levels of PK2 and PKR1 were modified under proinflammatory conditions, we next investigated the effect of PK2 on OA- and RA-SF under these conditions. We stimulated TNFα-pretreated OA- and RA-SF with 10^–11^ to 10^–14^ M PK2 and measured the cell culture medium concentration of the arthritis-aggravating factors IL-6 and MMP-3 and arthritis-inhibiting factors TIMP-1 and OPG (Fig. 6). PK2 strongly suppressed IL-6 secretion from OA-SF in a concentration-dependent manner, and the effect was antagonized by the PKR1-preferential antagonist PC-7 (Fig. 6A). PK2 mildly suppressed IL-6 secretion from RA-SF; however, the effect was not antagonized by PC-7. MMP-3 secretion from OA- and RA-SF was suppressed by PK2 in a concentration-dependent manner, and the effect was antagonized by PC-7 (Fig. 6B). TIMP-1 and OPG secretion from OA- and RA-SF were suppressed by PK2 in a concentration-dependent manner; however, the effect was not antagonized by PC-7 (Fig. 6C ,D).

We also assessed the antagonizing effect of PKRA7, which is a PKR1 and PKR2 antagonist. The results were similar to those obtained with PC-7, except the inhibitory effect of MMP-3 was not antagonized by PKRA7 in RA-SF (Supplemental Fig. S1). These data indicate that PK2 inhibits proinflammatory but not anti-inflammatory pathways, especially in OA-SF. PKR2 expression in OA-SF was particularly low after stimulation with TNFα, as shown in Figs. 3 and 4C. In addition, the effects of PC-7 and PKRA7 were similar. Therefore, the inhibitory effect on IL-6 and MMP-3 might be exerted through the PKR1 pathway but not the PKR2 pathway.

### PK2 does not affect the migration or proliferation of OA- and RA-SF

It was previously reported that PK2 regulates the migration and proliferation of various cells, including astrocytes^46^ and macrophages^31^. We examined cell migration using a scratch assay and cell viability using the MTT assay in OA- and RA-SF (Supplemental Fig. S2). PK2 at 10^–11^ M did not affect the cell migration or cell viability of OA- and RA-SF (Supplemental Fig. S2A,B).

### The anti-inflammatory effect of PK2 in OA-SF was mediated by NFκB signaling

**Figure 7:**
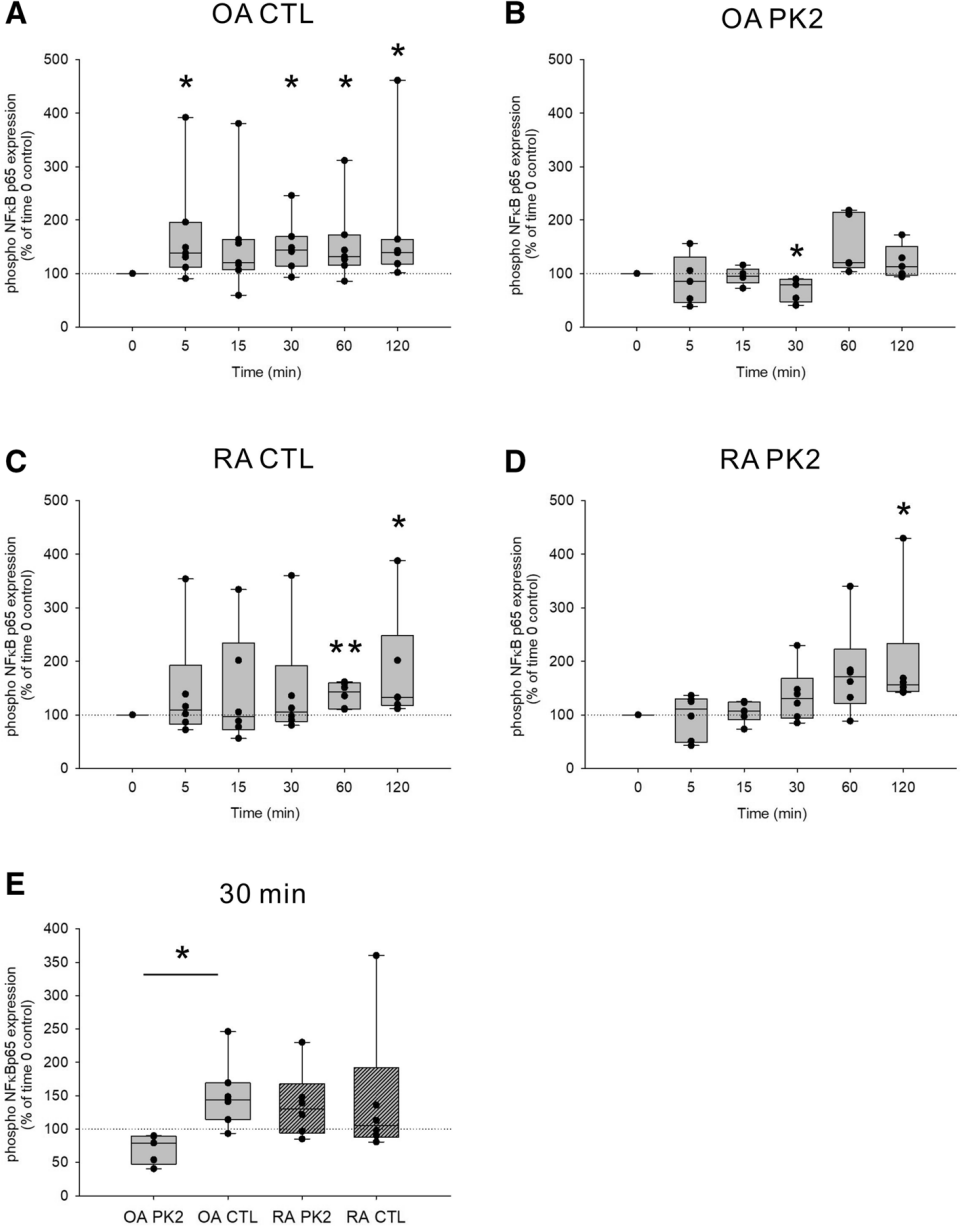
The induction of phospho-NFκB p65 by TNFα was attenuated in PK2 prestimulated OA-SF. Phospho-NFκB p65 protein in PK2 (10^–11^ M) or control (water) prestimulated OA- or RA-SF was examined by cell-based ELISAs 0, 5, 15, 30, 60, and 120 min after stimulation with TNFα (10 ng/ml). (**A**) Control prestimulated OA-SF. (**B**) PK2 prestimulated OA-SF. (**C**) Control prestimulated RA-SF. (**D**) PK2 prestimulated RA-SF. (**E**) Comparisons of phospho-NFκB p65 expression between PK2 or control prestimulated OA- or RA-SF 30 min after stimulation with TNFα. N = 5–8. Data were given as box plots, the description of which is given in the legend to Fig. 2. Each dot in the figure represents a different cell line derived from a different patient. For statistical analysis, the one-sample Wilcoxon signed-rank test was used for comparisons with time 0 as the control, representing the 100% line. In comparisons among the groups at 30 min after stimulation with TNFα (**E**), the Mann–Whitney test was used. *p < 0.05, **p < 0.01. *NFκB* nuclear factor kappa-light-chain-enhancer of activated B cells, *PK2* prokineticin 2, *CTL* control, *OA* osteoarthritis, *RA* rheumatoid arthritis, *SF* synovial fibroblasts, *TNFα* tumor necrosis factor α.

The production of IL-6 and MMP-3 was attenuated in TNFα-prestimulated OA-SF by PK2, as shown in Fig. 6 and Supplemental Fig. S1. Regarding the mechanism underlying the anti-inflammatory effect of PK2 in OA-SF, we hypothesized that PK2 inhibits signaling pathways downstream of TNFα^47^. To test this hypothesis, we examined the expression of phospho-NFκB p65 induced by TNFα in control or PK2 prestimulated OA- or RA-SF using cell-based ELISAs (Fig. 7). In control prestimulated OA-SF, phospho-NFκB p65 expression was upregulated at 5, 30, 60, and 120 min after TNFα stimulation (Fig. 7A). In contrast, in PK2 prestimulated OA-SF, phospho-NFκB p65 expression was not upregulated at any of the time points after TNFα stimulation and was downregulated at 30 min after stimulation (Fig. 7B).

In both control and PK2 prestimulated RA-SF, phospho-NFκB p65 expression was upregulated at 120 min after TNFα stimulation (Fig. 7C,D). Taken together, 30 min after TNFα stimulation, PK2 pretreatment significantly attenuated phospho-NFκB p65 expression in OA-SF but not RA-SF compared with the control pretreatment (Fig. 7E).

### PK2 mobilized granulocytes into joint spaces and promoted inflammation

As shown in Fig. 5A, the synovial fluid PK2 concentration in RA was higher than in OA. PK2 showed a chemotactic effect in PMN cells but not monocytes in vitro (Supplemental Fig. S3A). Generally, the number of PMN cells in RA synovial fluid is increased compared with OA synovial fluid^48^, and several reports have shown that PMN cells produce PK2^16,19,21,49^. We hypothesized that PK2 expressed in PMN cells mobilizes these cells into the synovial fluid. To test this hypothesis, we injected PK2 (range from 10^–9^ to 10^–11^ M) or PBS as the vehicle into healthy mouse knee joints and examined the degree of inflammation and cell infiltration.

After PK2 injection, we observed an increase in the knee circumference and extensive inflammatory cell infiltration into tissues (Supplemental Fig. S3B). Most localized cells were positive for the granulocyte marker Gr-1/Ly6G (Supplemental Fig. S3C) and negative for the macrophage marker F4/80 (Supplemental Fig. S3D). These findings correspond to the results of in vitro chemotaxis assays (Supplemental Fig. S3A). Taken together, our data indicated that PK2 mobilized PMN cells into joint tissues and caused inflammation.

## Discussion

For the first time, this study demonstrated a possible role of PK2 in OA and RA. The effect of PK2 was different depending on the cell type (SF and PMN cells) (Fig. 6, Supplemental Figs. S1 and S3). PK2 exhibited a chemotactic and proinflammatory effect on PMN cells (Supplemental Fig. S3), similar to previous reports^21,29,32^. Therefore, we predicted that PK2 would also have a proinflammatory effect on SF. However, PK2 exhibited an anti-inflammatory effect on TNFα-prestimulated SF (Fig. 6 and Supplemental Fig. S1), which appeared to be mediated by NFκB (Fig. 7). Moreover, the anti-inflammatory effect was diminished in RA-SF compared with OA-SF, and the anti-inflammatory activity of PK2 in OA-SF and the reduced anti-inflammatory effect of PK2 in RA-SF (Fig. 6 and Supplemental Fig. S1) were accompanied by an upregulation of PKR1 in TNFα-prestimulated OA-SF (Fig. 4). In contrast, PKR1 expression was downregulated in IL-1β-prestimulated RA-SF. These results were consistent with the difference in PKR1 expression between RA and OA synovial tissue under proinflammatory conditions in immunohistochemistry assays (Fig. 2).

**Figure 8:**
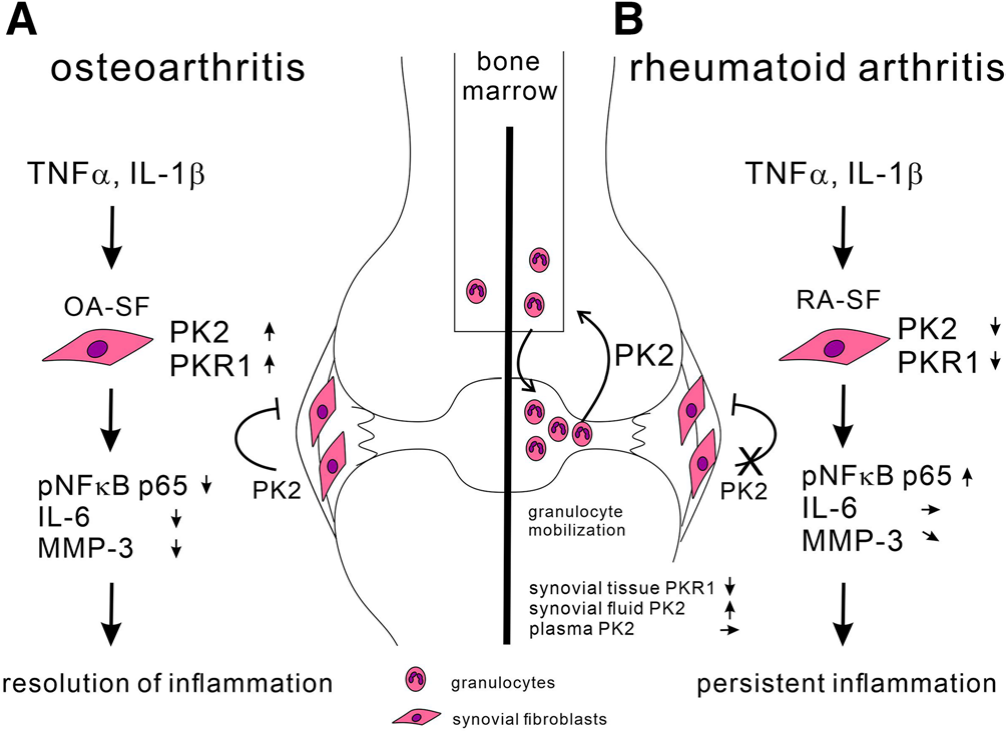
Model describing the dysregulation of endogenous inflammation by PK2 in arthritis. In OA, exposure to proinflammatory cytokines upregulates the expression of PKR1. PK2 secreted from OA-SF acts on PKR1 in an autocrine or paracrine manner and reduces the secretion of IL-6 and MMP-3 from OA-SF (**A**). In contrast, in RA-SF, exposure to proinflammatory cytokines downregulates the expression of PK2 and PKR1 (**B**). Therefore, PK2 exhibits a weaker anti-inflammatory effect in RA-SF compared with OA-SF. Indeed, the expression of PKR1 in RA synovial tissue was decreased compared with OA synovial tissue. This dysregulation in the endogenous inflammation-mediated modulation of PK2 in RA-SF may be associated with the chronicity of inflammation in the pathogenesis of RA. *PK2* prokineticin 2, *PKR1* prokineticin receptor 1, *OA* osteoarthritis, *RA* rheumatoid arthritis, *SF* synovial fibroblasts, *IL-1β* interleukin-1β, *TNFα* tumor necrosis factor α, *IL-6* interleukin-6, *MMP-3* matrix metalloproteinase 3, *pNFκB* phospho nuclear factor kappa-light-chain-enhancer of activated B cells.

This inverse regulation of PKR1 between RA-SF and OA-SF likely contributed to the differential effect of PK2 in RA- versus OA-SF exposed to inflammatory cytokines. Based on these results, we generated a model of PK2 functions in synovial tissue (Fig. 8). When OA synovial tissue is exposed to inflammatory cytokines, including IL-1β and TNFα, in specific clinical situations, such as systemic inflammation, injury, and intense exercise, inflammation caused by these factors often resolves spontaneously. In these situations, PK2 might be a critical factor responsible for the inhibition of inflammation (Fig. 8A).

In contrast, RA synovial tissue is continuously exposed to inflammatory cytokines, leading to the downregulation of PK2 and PKR1 in RA-SF. As a result, the endogenous inflammation-mediated modulation of PK2 is impaired, and inflammation persists in RA synovial tissue (Fig. 8B). Taken together, this dysregulation in the endogenous inflammation-mediated modulation of PK2 and its receptor PKR1 in RA-SF may partially explain the chronicity of inflammation in the pathogenesis of RA.

To understand the inflammation-mediated modulation system of PK2 in OA-SF, we first determined which of the two receptors are associated with the anti-inflammatory effect of PK2 in these cells. Our results showed that PKR1 but not PKR2 was expressed in unstimulated OA-SF (Fig. 3), and no change in the PKR2 expression level was observed following TNFα or IL-1β stimulation (Fig. 4). Therefore, PKR2 expression was thought to be substantially lower than PKR1 expression. In addition, the effects of the PKR1-preferential antagonist PC-7 (Fig. 6) and PKR1 and PKR2 antagonist PKRA7 (Supplemental Fig. S1) were similar. Based on these results, we suggest that the anti-inflammatory effect of PK2 on OA-SF is likely mediated through the PKR1 pathway but not the PKR2 pathway.

Second, we investigated how PKR1 downstream signaling influences the anti-inflammatory pathway. We demonstrated that PK2 inhibited NFκB signaling, one of the proinflammatory downstream signaling pathways in OA-SF (Fig. 7). However, the mechanism by which PKR1 downstream pathways inhibit NFκB signaling remains unknown. PKR1 couples to Gαq, Gαs, and Gαi proteins, and several reports of anti-inflammatory effects via G protein-coupled receptors have been described. For instance, Gαq-coupled receptors activate AMPK^50^. The activation of AMPK suppresses the inflammatory response in SF^51,52^ and rapidly inhibits TNFα-stimulated IKK/IκB/NFκB signaling in adipocytes^53^. Regarding Gαs-coupled receptors, an increase in cAMP suppresses proinflammatory responses via the PKA/CREB pathway in different cell types^53–56^. Therefore, we speculated that one or multiple G-protein pathways might be associated with the inhibition of NFκB phosphorylation. Recently, Szatkowski et al. reported that PKR1 suppressed preadipocyte proliferation and differentiation, and macrophages infiltrated adipose tissue in adipocyte/preadipocyte-specific PKR1 knockout mice^57^. This report also indicates the anti-inflammatory effect of PKR1 and supports our results. However, no study has reported the mechanism underlying the anti-inflammatory effect of PKR1 downstream signaling pathways to date. Thus, further investigations to address these issues are warranted.

Determining how PK2 contributes to the development of arthritis is challenging for two reasons. First, the expression of PKRs varies depending on the microenvironment and cell type^15,58^. In the present study, we demonstrated the presence of PKR1 but not PKR2 in SF from OA and RA patients (Fig. 3). In human synovial tissue, both PK2- and PKR1-positive cells and PKR2-positive cells were identified (Fig. 2). The presence of PKR2-positive cells shows that PK2 acts on both PKR1 and PKR2 in human synovial tissue because the affinities of PKR1 and PKR2 are similar^13^. Furthermore, the expression of both receptors is altered by different microenvironmental conditions, including exposure to inflammatory cytokines, as shown in this study (Fig. 4). However, the role of PKR2-expressing cells is not clear.

Previously, we demonstrated that PKR1 and PKR2 were expressed in mice with CIA, and the severity of arthritis was correlated with the expression level of PKR2 rather than PKR1^37^. In mice with CIA, PKR2-positive cells were macrophage-like cells, not fibroblasts^37^. Moreover, PKRA7 (a PKR1 and PKR2 antagonist; IC_50_ = 5 nM and 8.2 nM for PKR1 and PKR2, respectively) suppressed the severity of arthritis in the same model^37^. This was in contrast to the anti-inflammatory effect of PK2 on SF via PKR1 in the present study. Given these points, PKR2 in macrophage-like cells might have a stronger proinflammatory effect compared with the anti-inflammatory effect of PKR1 in SF during the development of arthritis.

We showed that PKR2 expression was clearly induced by TGFβ in OA- and RA-SF (Fig. 4). TGFβ has a pro- or anti-inflammatory effect depending on the microenvironment. For instance, TGFβ promotes the production of extracellular matrix proteins, such as type II collagen and aggrecan, in chondrocytes and has protective effects on cartilage, whereas it causes cartilage degradation when expressed with proinflammatory cytokines, including IL-1 and TNFα^59^. In addition, TGFβ functions as an anti-inflammatory factor by inducing regulatory T cell differentiation and a proinflammatory factor by promoting Th17 cell differentiation when present with IL-6^60^. Thus, the microenvironment dictates the pro- or anti-inflammatory activity of a cytokine. The effect of PKR2 induced by TGFβ might also be modified according to the local environmental factors. Further exploration of the role of PKR2-expressing cells in synovial tissue is needed to elucidate the function of PK2 in arthritis.

Second, the cell types contributing to arthritis vary during different phases of the disease. Generally, granulocytes are increased in the synovial fluid of patients with active early arthritis^48^ and are the main source of PK2^16,19,21,49^. Our results showed that PK2 injection into the joints of mice induced granulocyte migration and promoted inflammation at the injection sites (Supplemental Fig. S3), and the concentration of PK2 in RA synovial fluid was higher than that in OA synovial fluid (Fig. 5). Considering these facts, we speculate that the granulocytes in RA synovial fluid produce PK2 and that PK2 secreted from granulocytes mobilizes these cells into the synovial fluid and induces inflammation. This phenomenon may occur in the synovial tissue and synovial fluid of acute arthritis models, including RA (early phase)^61^, crystal-induced arthritis^62^, infectious arthritis^62^, mouse CIA (early phase)^38^, and collagen antibody-induced arthritis^38^, as most of the influential cells in these arthritides are granulocytes.

The anti-inflammatory effect of PKRA7 in mouse CIA^37^ might be due to the suppressed migration of granulocytes from the bone marrow during the early phase of arthritis. Moreover, infiltrating granulocytes are reduced in the synovial tissue of chronic arthritis models compared with acute arthritis^63^. As a result, the main source of PK2 in synovial tissue changes from granulocytes to synovial cells, including SF, synovial macrophages, and inflammatory cells (except for granulocytes). It has been suggested that PK2 acts locally on synovial cells at a very low concentration in an autocrine or paracrine manner but not an endocrine manner because the concentration of superfusate was lower than that of synovial fluid and plasma. In addition, PK2 reduces the secretion of IL-6 from SF. This may occur in chronic arthritis, including the chronic phase of RA, OA, and mouse CIA. Therefore, PK2 has an ambivalent effect in arthritis that is dependent on the effector cell type, phase of disease development, and microenvironment in the presence or absence of additional cytokine stimulators.

This study has some limitations. First, we did not examine the effect of PKR1 knockout or overexpression in TNFα-prestimulated OA-SF or RA-SF, respectively. This experiment might directly demonstrate the effect of PK2 because of the alteration in PKR1 expression in SF. Second, we did not identify the cell types positive for PK2, PKR1, and PKR2 in arthritic synovial tissue using immunohistochemistry. These points should be further investigated to better understand the role of the PK2-PKR system during the development of RA.

In conclusion, we demonstrated that PK2, PKR1, and PKR2 were expressed in synovial tissue. This indicates that PK2 acts locally. Indeed, PK2 had an anti-inflammatory effect on OA-SF that was likely mediated through the PKR1 pathway, whereas this anti-inflammatory effect was attenuated in RA-SF due to the alteration in PKR1 expression. This study provides a new model to explain some aspects regarding the chronicity of inflammation in RA. However, the effect of PK2 in the synovial tissue may vary depending on the effector cell type or receptor expression. For this reason, explaining the effect of PK2 on arthritis remains challenging.

## Supplementary Information

Supplementary Information 1.

Supplementary Information 2.

Supplementary Information 3.

Supplementary Information 4.

## Data Availability

The datasets generated and analyzed for the present study are available from the corresponding author on reasonable request.
