# Supplementary material for: Differential inflammation-mediated function of prokineticin 2 in the synovial fibroblasts of patients with rheumatoid arthritis compared with osteoarthritis
Source: Sci Rep. 2021 Sep 15;11:18399. doi: 10.1038/s41598-021-97809-z (PMC8443611; doi:10.1038/s41598-021-97809-z)
Supplement: Supplementary file 1 — Supplementary Information 1. [file 41598_2021_97809_MOESM1_ESM.docx]

**RESEARCH ARTICLE - Supplemental methods and figures**

**Differential inflammation-mediated function of prokineticin 2 in the synovial fibroblasts of patients with rheumatoid arthritis compared with osteoarthritis**

Kentaro Noda^1,2*^, Bianca Dufner^1^, Haruyasu Ito^2^, Ken Yoshida^2^, Gianfranco Balboni^3^, Rainer H. Straub^1^

^1^Laboratory of Experimental Rheumatology and Neuroendocrine Immunology, Department of Internal Medicine I, University Hospital Regensburg, Regensburg, Germany

^2^Division of Rheumatology, Department of Internal Medicine, The Jikei University School of Medicine, Tokyo, Japan

^3^Department of Life and Environmental Sciences, University of Cagliari, Cagliari, Italy

^*^Corresponding author

Address correspondence to Kentaro Noda, MD, Ph.D., Laboratory of Experimental Rheumatology and Neuroendocrine Immunology, Department of Internal Medicine I, University Hospital Regensburg, Biopark I, Am Biopark 9, DE–93053 Regensburg, Germany, e-mail: [knoda3353@jikei.ac.jp](mailto:knoda3353@jikei.ac.jp)

**Supplemental methods**

**MTT assay.** For the MTT assay, 1 × 10^4^ cells per well were seeded into 96-well plates. The next day, cells were prestimulated with TNFα (10 ng/ml) for 48 h and then stimulated with PK2 (10^-11^ M) and PC-7 (1 μM) for 24 h. After stimulation, cell viability was measured using the MTT Cell Proliferation Assay Kit (3942B) from Boster biological technology, Pleasanton, CA, USA, following the manufacturer’s protocol.

**Migration scratch assay.** For migration scratch assays, 2 × 10^5^ cells per well were seeded into 12-well plates. The next day, cells were prestimulated with TNFα (10 ng/ml) for 48 h. A single scratch wound was made in the middle of each well using a 200-μl sterile tip, and then the cells were washed twice with phosphate buffer saline (PBS). Subsequently, cells were incubated with PK2 (10^-11^ M) and PC-7 (1 μM) for 24 h. The wounds were photographed at 0 and 24 h after stimulation with PK2 in 4 different fields under an inverted microscope (Primovert, Carl Zeiss, Göttingen, Germany). The pictures were analyzed by Image J, and the percent of the area closed was measured.

***In vitro* monocyte and polymorphonuclear (PMN) cell chemotaxis assays.** Monocytes and PMN cells were isolated from healthy human peripheral blood. We performed chemotaxis assays with monocytes and PMN cells cultured in the presence of PK2 (range from 10^-7^ M to 10^-16^ M) using Boyden chambers as described previously^1^. PBS was used as a negative control. Assays were performed in quadruplicate, and the number of migrated cells was counted in each replicate well in 3 high power fields of view (×400). The results were expressed as the fold increase compared with PBS. We performed this study according to the Helsinki Declaration of 1975, as revised in 1983. Approval for this study was obtained from the Ethics Committee of the Jikei University School of Medicine (approval number 25-268). All participants understood the purpose of the study and provided informed consent.

**Inflammatory arthritis mouse model.** Five-week-old DBA1/J mice (n = 40) were purchased from Sankyo Labo Service (Tokyo, Japan). The mice were divided into a PBS group and a PK2 treatment group (range from 10^-9^ to 10^-11^ M). The mice were anesthetized, and the knee circumference was determined by caliper measurements before intraarticular injection. The anesthetized mice received 20 µl/knee joint of PBS or PK2. The knee circumference measurements were taken in a blinded manner before and 24 h after the intraarticular injection for all mice. The knee joints were isolated, fixed, and embedded in paraffin as described previously^1^. The block was sectioned into 4-mm slices and used for hematoxylin and eosin (HE) staining and immunohistochemistry. For the analysis of knee joints harvested from the inflammatory arthritis mouse model, HE and immunohistochemical staining were performed on paraffin sections as described previously^1^. A rat anti-mouse Gr-1/Ly6G monoclonal antibody (550291, 312.5 pg/ml, BD Biosciences, Heidelberg, Germany) for the analysis of PMN cells and a rat anti-mouse F4/80 monoclonal antibody (MCA497RT, 2 mg/ml Bio-Rad, München, Germany) for the analysis of monocytes/macrophages were used as primary antibodies. The sections were examined under a microscope (Axio Imager A1, Carl Zeiss, Göttingen, Germany). Then, the number of Gr-1/Ly6G -and F4/80-positive cells in the joint space where the injections were administered was calculated as the average of the number of cells in 3 high-power fields of view. The protocol for animal experiments was reviewed and approved by the Institutional Animal Care and Use Committee of the Jikei University (approval number 25-046) and conformed to the Guidelines for the Proper Conduct of Animal Experiments of the Science Council of Japan (2006).

**Supplemental reference**

1. Yoshida, K. *et al.* Citrullination of Epithelial Neutrophil-Activating Peptide 78/CXCL5 Results in Conversion from a Non-Monocyte-Recruiting Chemokine to a Monocyte-Recruiting Chemokine. *Arthritis Rheumatol.* **66**, 2716–2727 (2014).

**Supplemental figure legends**

Supplementary figure 1. Influence of PK2 and PKRA7 (or DMSO) on IL-6 (A), MMP-3 (B), TIMP-1 (C), and OPG (D) production from TNFα−prestimulated OA- and RA-SF.

The dotted line indicates the control level of 100% (TNFα without PK2). All data are given as the median (25th percentile, 75th percentile). N = 8. For comparisons between the DMSO and PKRA7 groups, a two-way ANOVA followed by the Bonferroni post hoc test was used (*p<0.05, **p<0.01). For comparisons with a control level of 100%, the one-sample Wilcoxon signed-rank test was used (compared with the control in the PKRA7 group: §, p<0.05 and §§, p<0.01; compared with the control in the DMSO group: †p<0.05 and ††p<0.01). Abbreviations: PK2, prokineticin 2; DMSO, dimethylsulfoxide; OA, osteoarthritis; RA, rheumatoid arthritis; SF, synovial fibroblasts; IL-6, interleukin-6; TNFα, tumor necrosis factor α; MMP-3, matrix metalloproteinase 3; TIMP-1, tissue inhibitor of metalloproteinase 1; OPG, osteoprotegerin; NS, not significant; ANOVA, analysis of variance.

Supplementary figure 2. PK2 did not affect the viability or migration of OA- and RA-SF.

The cell viability and cell migration of TNFα−prestimulated OA- or RA-SF after stimulation with PK2 (10^-11^ M) and DMSO or PC-7 (1 μM) were assessed by the MTT assay (A) and migration scratch assay (B), respectively. Data were shown as box plots, in which the boxes demonstrate the 25th and 75th percentiles, the lines within the boxes demonstrate the median, and the lines outside the boxes demonstrate the 10th and 90th percentiles. The dotted line indicates the control level of 100% (TNFα without PK2). N = 4-5. For statistical analysis, the Mann–Whitney test was used. Abbreviations: PK2, prokineticin 2; OA, osteoarthritis; RA, rheumatoid arthritis; SF, synovial fibroblasts; DMSO, dimethylsulfoxide; TNFα, tumor necrosis factor α; OD, optical density; MTT, 3-(4,5-dimethylthiazol-2-yl)-2,5-diphenyltetrazolium bromide.

Supplementary figure 3. PK2 recruited PMN cells *in vitro* and induced neutrophil-driven inflammatory arthritis.

(A) The chemotaxis of PMN cells and monocytes obtained from healthy humans in response to PK2 (range from 10^-7^ to 10^-16^ M) was measured using Boyden chambers. The results were expressed as the fold increase compared with PBS. The dotted line indicates the control level of a 1-fold increase (PBS). All data are given as the median (25th percentile, 75th percentile). N = 4. For comparisons with the control level (1-fold increase), the one-sample Wilcoxon signed-rank test was used (*p<0.05, **p<0.01). (B) HE staining showed that the number of inflammatory cells in the synovium was higher in mice injected with PK2 at 10^-10^ M (left panel: ×400) compared with PBS (middle panel: ×400). The knee circumference was significantly increased in mice injected with PK2 (range from 10^-9^ to 10^-11^ M) compared with PBS (right panel). N = 10. (C and D) Immunohistochemical staining for PMN cells (C) (Gr-1/Ly6G) and monocytes (D) (F4/80) was performed using the knee joints from the inflammatory arthritis mouse model. N =20. Gr-1/Ly6G- and F4/80-positive cells were stained brown. (C) Gr-1/Ly6G-positive cells in the knee joint of the mice injected with PBS (left panel: ×400). Gr-1/Ly6G-positive cells in the knee joint of mice injected with PK2 at 10^-10^ M (middle panel: ×400). (middle panel) The fold increase in Gr-1/Ly6G-positive cells was significantly higher in the knee joints of mice injected with PK2 at 10^-10^ M compared with PBS. (D) F4/80-positive cells in the knee joint of mice injected with PBS (left panel: ×400). F4/80-positive cells in the knee joint of mice injected with PK2 at 10^-10^ M (middle panel: ×400). There was no significant difference in the fold increase in F4/80-positive cells between both groups (right panel). Data for histopathological analysis were presented as box plots, in which the boxes demonstrate the 25th and 75th percentiles, the lines within the boxes demonstrate the median, and the lines outside the boxes demonstrate the 10th and 90th percentiles. For statistical analysis, the Mann–Whitney test was used. *p<0.05, **p<0.01.
