# Supplementary figures and images for: Differential inflammation-mediated function of prokineticin 2 in the synovial fibroblasts of patients with rheumatoid arthritis compared with osteoarthritis
Source: Sci Rep. 2021 Sep 15;11:18399. doi: 10.1038/s41598-021-97809-z (PMC8443611; doi:10.1038/s41598-021-97809-z)

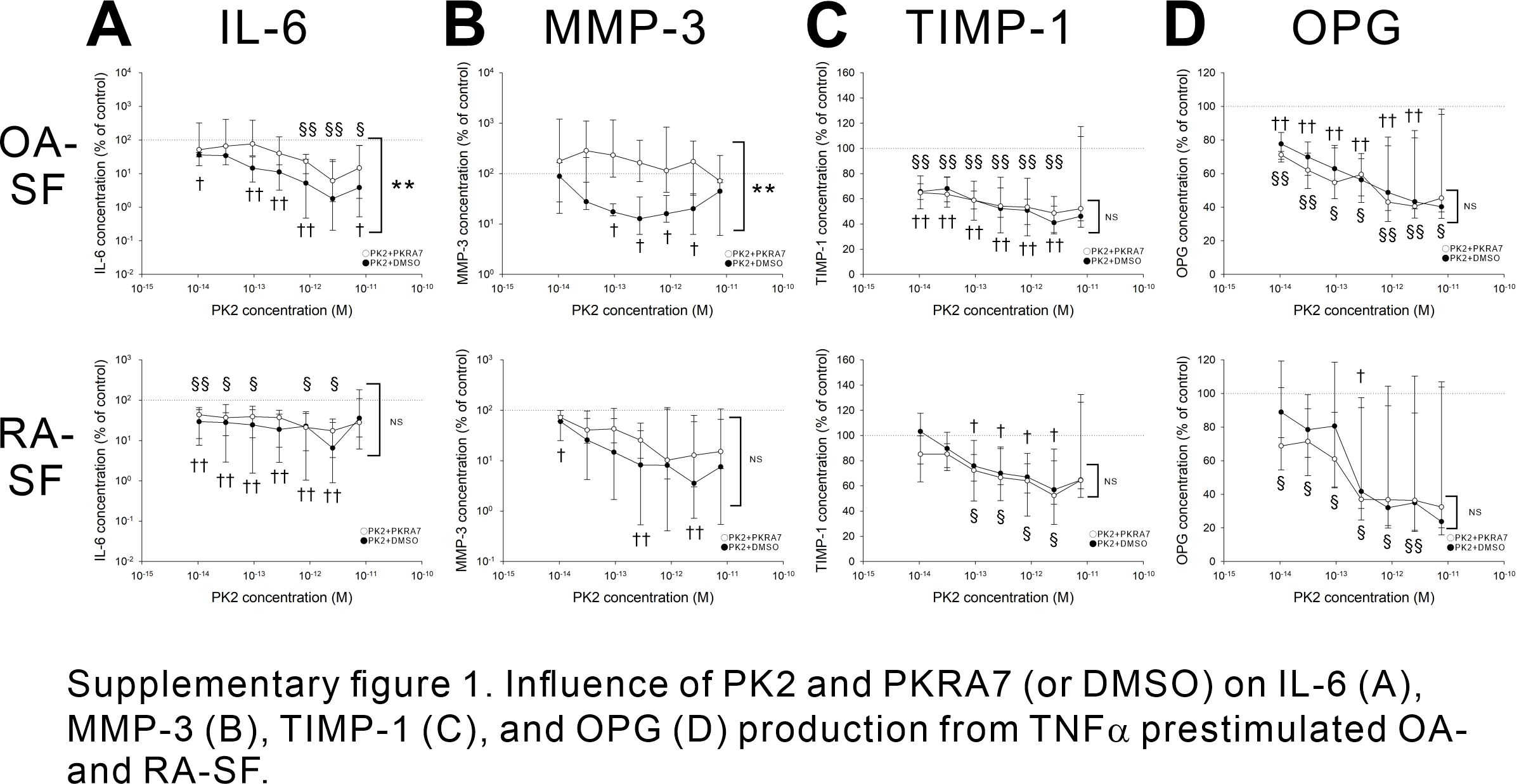

Supplement: Supplementary file 2 — Supplementary Information 2. [file 41598_2021_97809_MOESM2_ESM.jpg]

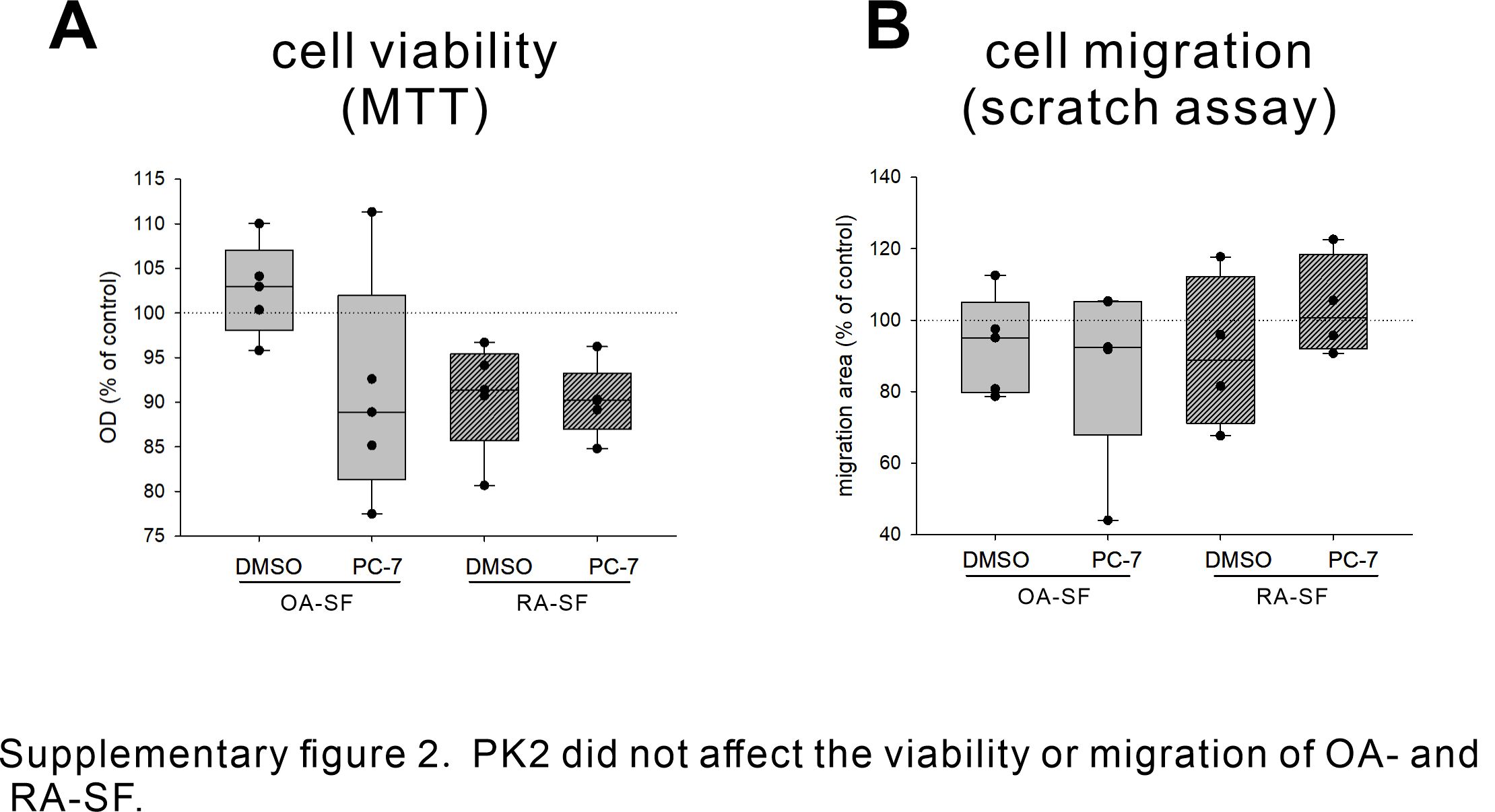

Supplement: Supplementary file 3 — Supplementary Information 3. [file 41598_2021_97809_MOESM3_ESM.jpg]

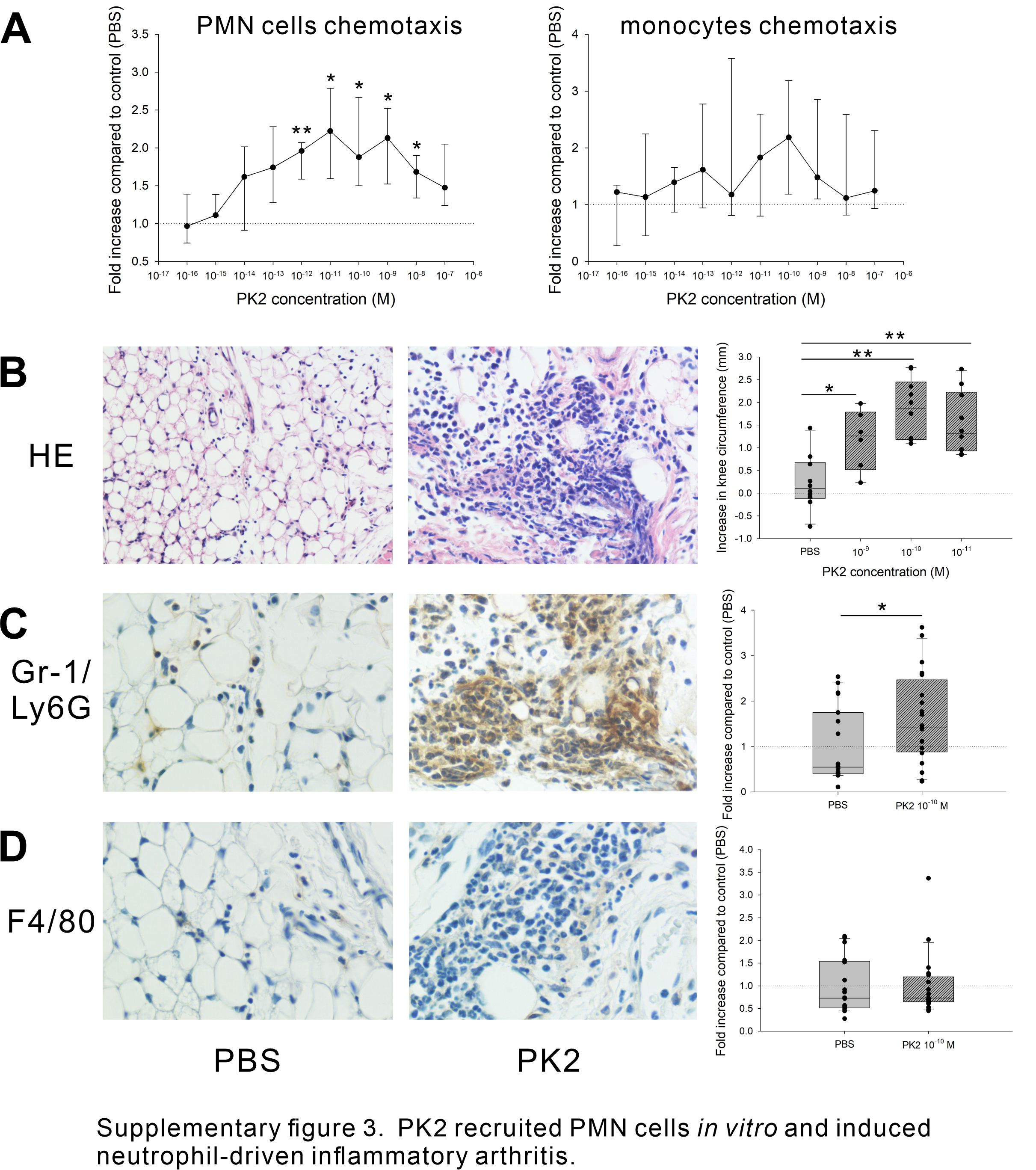

Supplement: Supplementary file 4 — Supplementary Information 4. [file 41598_2021_97809_MOESM4_ESM.jpg]
